# A Review of the Prospera Spinal Cord Stimulation System with Multiphase Stimulation and Proactive Care

**DOI:** 10.1007/s11916-024-01318-3

**Published:** 2025-01-13

**Authors:** Ramana K. Naidu, Leonardo Kapural, Sean Li, Caitlin Tourjé, Joseph Rutledge, David Dickerson, Timothy R. Lubenow

**Affiliations:** 1 MarinHealth Spine Institute, a UCSF Affiliate, 2 Bon Air Rd #120, Larkspur, CA 94939 USA; 2 Carolinas Pain Institute, Winston-Salem, NC USA; 3 Premier Pain Centers (an affiliate of National Spine and Pain Centers), Shrewsbury, NJ USA; 4 Spanish Hills Interventional Pain Specialists Inc, Camarillo, CA USA; 5 Center for Pain Management, Lafayette, IN USA; 6 Department of Anesthesiology, Critical Care and Pain Medicine, Endeavor Health, Evanston, IL USA; 7 Pritzker School of Medicine, University of Chicago, Chicago, IL USA; 8 Department of Anesthesiology, Rush University Medical Center, Chicago, IL USA

**Keywords:** Spinal cord stimulation, Multiphase stimulation, Remote monitoring, Remote programming, Proactive care, Chronic pain

## Abstract

**Purpose of Review:**

The purpose of this review is to describe the development and key features of the Prospera™ Spinal Cord Stimulation (SCS) System, as well as the clinical evidence supporting its use. Prospera delivers therapy using a proprietary multiphase stimulation paradigm and is the first SCS system to offer proactive care through automatic, objective, daily, remote device monitoring and remote programming capabilities.

**Recent Findings:**

Results from the recently published BENEFIT-02 trial support the short-term safety and efficacy of multiphase stimulation in patients with chronic pain. BENEFIT-03 is an ongoing, multicenter, single-arm study with 24-month follow-up; interim analyses suggest that multiphase therapy is safe and effective and that patients and clinicians have positive experiences with remote device management.

**Summary:**

Preliminary evidence suggests that the Prospera SCS System represents an opportunity to improve patient care by combining an effective multiphase stimulation paradigm with an efficient proactive care model.

## Introduction

Chronic pain affects approximately 50 million people in the US and is associated with lost work productivity, reduced quality of life, and high healthcare costs [1, 2]. Spinal cord stimulation (SCS) was developed in the 1960s and has been used for decades as a safe and effective option to manage a variety of pain conditions, such as persistent spinal pain syndrome and complex regional pain syndrome [3]. Traditional, or tonic, SCS involves the delivery of electrical stimulation to the spinal cord that produces a tingling sensation, known as paresthesia, which some patients describe as unpleasant [3]. Recently, various sub-perception SCS waveforms were developed (eg, high frequency, burst) that provided effectiveness in reducing chronic pain intensity in clinical trials [4–10]. Despite these advances, clinical challenges (eg, access to care, loss of efficacy, lead migration, need for magnetic resonance imaging [MRI]) persist in real-world settings and may contribute to high rates of device explantation [11–17]. Some of these challenges could be addressed via prompt issue identification and resolution, enabled by recent developments in remote SCS device management [18], though further research is needed.

This article reviews an SCS system (Prospera™, BIOTRONIK SE & Co. KG, Berlin, Germany) that was approved by the U.S. Food and Drug Administration in March 2023 for the management of chronic pain in the trunk and/or limbs. Prospera is currently the sole SCS system with a proprietary multiphase stimulation paradigm (RESONANCE™) and a remote device management platform (Embrace One™) that offers proactive care via automatic, objective, daily, remote device monitoring and on-demand remote programming capabilities. The goal of this review is to describe the development history, key technical features, and the evidence supporting this novel stimulation paradigm and its efficient delivery through proactive remote management.

## Key Features of the Prospera SCS System

### Development of Multiphase Stimulation

The Prospera SCS system features a proprietary multiphase stimulation paradigm (RESONANCE) that was designed to address therapy limitations due to known challenges associated with existing SCS therapy designs. Evidence suggests that minimizing the electrical dose delivered to the spinal cord may provide clinically meaningful pain relief and reduce side effects [19]. However, recent stimulation patterns involve the delivery of concentrated electrical stimuli to a relatively small area of the spinal cord. A proprietary multiphase dosing approach was designed to distribute a lower charge by leveraging the temporal and spatial summation effects of the central nervous system, thus activating a larger area of the spinal cord with lower energy demands and potentially reducing charging burden and risk of overstimulation. This broad vertebral coverage additionally allows for pain relief that is resistant to lead displacement or postural changes. Additionally, multiphase stimulation was designed to have low-complexity programming requirements, alleviating the need for programming "experts" and allowing for an improved and more standardized patient experience despite differences in local field representatives.

**Fig. 1:**
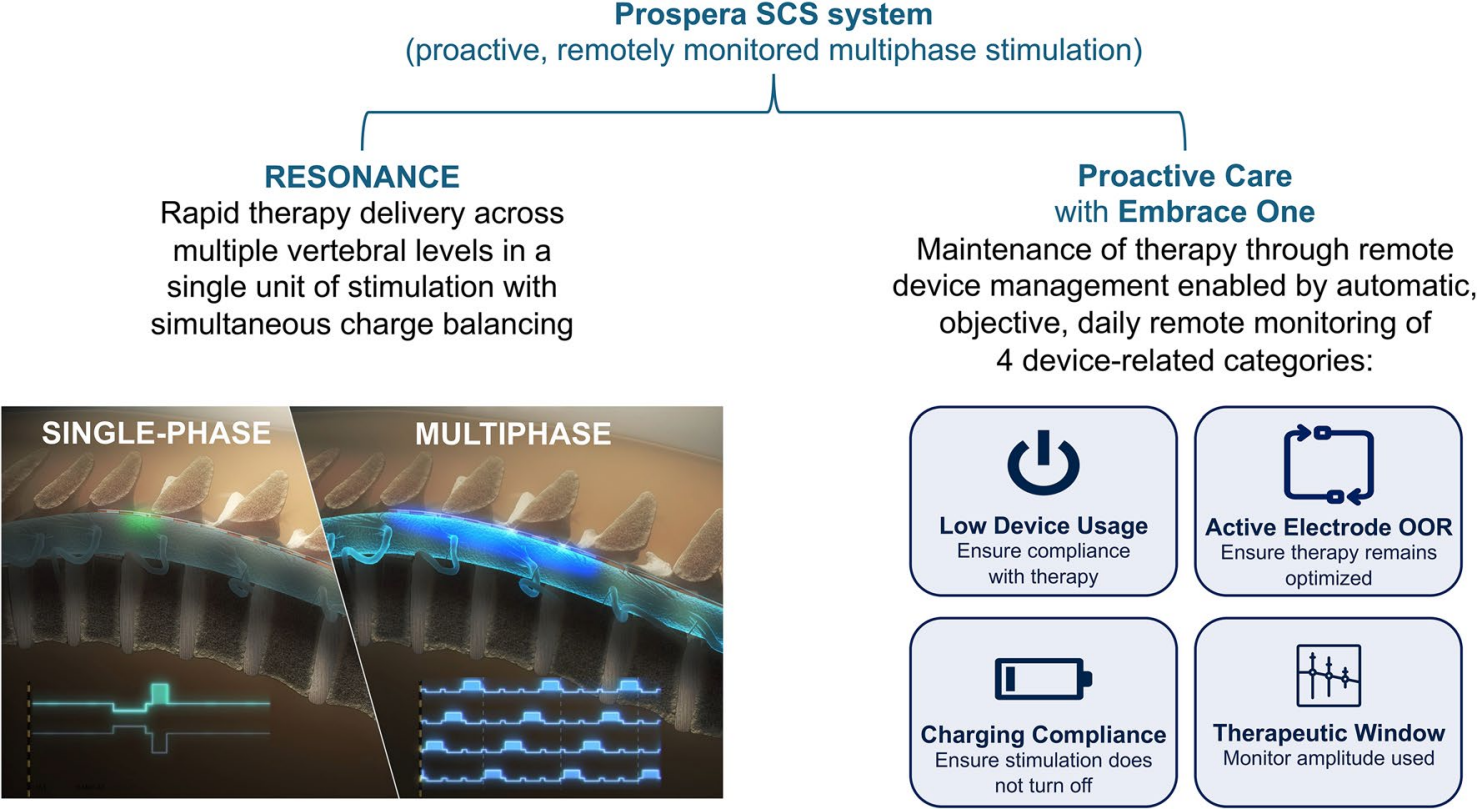
Graphic representation of RESONANCE multiphase stimulation (BIOTRONIK SE & Co. KG, Berlin, Germany) versus single-phase stimulation and proactive care enabled by remote device monitoring and remote programming. Inset image: © 2023, courtesy of BIOTRONIK. OOR, out of range; SCS, spinal cord stimulation

Compared with single-phase stimulation, the proprietary stimulation paradigm uses a characteristic multiphase stimulation, which provides broader coverage by sweeping therapeutic micropulses across multiple electrodes. A key distinguishing feature of multiphase stimulation is the charge balancing, an important aspect of all neuromodulation systems. Prospera’s BioARC™ stimulation engine performs charge balancing of each electrode with the anodic return currents from other electrode’s stimulation phases. In contrast, currently available single-phase SCS systems must interrupt therapy delivery for charge balancing to prevent electrode and/or tissue damage. With the BurstDR™ waveform (Abbott, Austin TX), the rebalancing occurs passively and requires a certain amount of time during the quiescent phase following a burst. Likewise, with traditional tonic stimulation, rebalancing occurs passively and must allow a minimum time between pulses for this to occur. This new multiphase stimulation paradigm instead balances charge for each electrode during the therapeutic stimulation phases of other adjacent electrodes so that therapeutic micropulses are rapidly rotated with a short (0.3 ms) pulse width typically over a tripolar or quadripolar electrode configuration. This proprietary method allows therapy to be delivered across multiple vertebral levels in a continuous pattern during every phase (Fig. 1) [10].

Development of RESONANCE was driven by a series of preclinical and clinical studies. The BENEFIT-01 study was designed to evaluate the influence of clinically relevant SCS parameters on patient perception by creating a parametric map of perception threshold and distributions [20]. The study was conducted in the US at 7 clinical centers following institutional review board approval and aimed to evaluate the following: effect of various SCS parameters on paresthesia threshold, comfort, and quality; effect of electrode configuration on efficiency. Patients with low back and/or leg pain who had completed a percutaneous SCS trial with a commercial system were enrolled (n = 43). Prior to removal of the commercial trial leads, participants received approximately 90 min of test stimulation using varied SCS parameters (amplitude, frequency, pulse width, electrode configuration, cycling and multifrequency stimulation). Results indicated that pulse width, rather than frequency, primarily influenced the paresthesia threshold and that pulse frequency primarily influenced paresthesia quality [20]. A frequency range of about 60 Hz to 2.4 kHz was associated with the greatest level of paresthesia comfort. Further, a wider-spaced tripolar stimulation configuration was more energy efficient than tightly-spaced configurations [20].

Results from BENEFIT-01 were applied in a computational study that modeled the effects of multiphase stimulation versus single-phase therapies [21]. Modeling results estimated that, compared with single-phase SCS therapies, multiphase stimulation provided 2 to 5 times greater therapeutic coverage and used 30–60% less power. This modeling study, along with the BENEFIT-01 study results, laid the foundation for the technical development of RESONANCE therapy.

Functional ultrasound (fUS) techniques were used in preclinical work to quantify the expected response within the dorsal horn to multiphase stimulation. fUS is an emerging imaging technique that monitors the brain or spinal cord for local hemodynamic changes that are tightly coupled to neural activity [22, 23]. Previous studies in animal pain models and humans have demonstrated that fUS can be used to measure activation in the spinal cord with high spatiotemporal resolution, including during SCS therapy [24, 25]. Preliminary fUS results from 5 large animal (ovine) models indicated that hemodynamic responses to multiphase stimulation covered a larger area of the spinal cord with greater depth than other tested stimulation modes, supporting the broad therapeutic coverage achieved with multiphase stimulation [26].

### System Technical Features

The multiphase SCS system includes a rechargeable implanted pulse generator, one or more percutaneously implantable leads, a clinician programmer, a trial stimulator, a patient programmer, a charger, and other standard accessories [27]. Two 8-electrode leads deliver electrical stimulation using one of 12 programs with any combination of up to 4 cathodes and 4 anodes or, in the case of multiphase stimulation mode, 3–4 electrodes which act as both anodes and cathodes. Frequencies range from 2 to 1400 Hz.

### MRI Conditionality

It is estimated that 84% of patients implanted with SCS devices require at least 1 MRI within 5 years of implant [28]. The Prospera SCS System is labeled as head, extremity, and full body MRI conditional at 1.5T and 3T in normal operating mode without exclusion zones, making it the first available SCS system approved for 3T full body scanning. MRI mode can be enabled by the clinician programmer or patient programmer. Remote management capabilities (described in the next section) can be used to confirm MRI conditions and MRI mode. Pre- and post-MRI device checks can also be conducted remotely.

### Remote Device Management and Proactive Care

Most commercially available SCS systems require in-person visits for routine follow-up and therapy optimization. These visits stress clinic resources and can be time-consuming and burdensome to patients and their caregivers; real-world data suggest that it may take > 7 days for SCS issues to be resolved in a traditional, in-person setting [29, 30]. Prolonged wait times and gaps in therapy may negatively impact health-related quality of life and overall pain management. Advancements in SCS technology have enabled remote device management, which provides an opportunity to optimize stimulation use from a distance, rapidly identify and address SCS-related issues, and improve the patient and clinician SCS experience, potentially leading to enhanced therapy outcomes [18]. Remote device management is a broad term that encompasses both remote monitoring (diagnostic) and remote programming (therapeutic) [18]. Data gathered via remote monitoring can be collected automatically by the device or self-reported by the patient and/or caregiver. Device-collected data can be further categorized as either device-related or physiologic (eg, pedometry). Remote programming occurs as needed and involves adjustment to the operating parameters of the SCS system to meet patient needs.

Prospera is the first SCS system to offer automatic, objective, daily transmission of device data for the purposes of remote monitoring along with the capability to perform therapeutic adjustments and remote programming utilizing either cellular or Wi-Fi connectivity. Through the Embrace One proactive care platform, device-related data are securely transmitted for remote monitoring of system use/performance and processed to generate proactive notifications across four clinically actionable metrics: low device usage, amplitude outside of expected therapeutic range, charging compliance, and active electrode impedance out of range (Fig. 1). The thresholds used to trigger proactive notifications can be customized for individual patients. Additionally, the remote care team monitors data transmission throughout the course of therapy and intervenes with the patient as needed (eg, in the case of missing data transmission). All diagnostic data are available in a secure, centralized portal for review by healthcare providers and manufacturer representatives to support optimization and maintenance of SCS. In addition, patients or caregivers can enter subjective metrics (eg, pain intensity, sleep quality) through a smartphone app allowing healthcare professionals to gain additional insight into the effectiveness of a patient’s therapy. Remote device management is intended to rapidly identify and address SCS device-related issues to optimize the SCS patient and clinician experience.

### Available Clinical Evidence

The safety and effectiveness of multiphase stimulation has been assessed in BENEFIT-02 and BENEFIT-03, the latter of which is ongoing to evaluate the Prospera SCS system with remote device management and proactive care (Table 1).

**Table 1:** Summary of Clinical Effectiveness Studies of Multiphase Stimulation

Study	**BENEFIT-02 (NCT03594266)**	**BENEFIT-03 (NCT04683718)**
Objective	To assess the safety and comparative efficacy of 2 multiphase SCS therapies following a trial period with a commercially available SCS system	To evaluate the long-term efficacy and safety of the Prospera SCS System with multiphase stimulation (RESONANCE), and automatic daily transmission of objective device monitoring data with remote programming
Study design	Prospective, multicenter, randomized, parallel-assignment, single-blind feasibility study conducted at 16 US sites with IRB approval Eligible participants who experienced a successful trial with a commercially available SCS system were randomized to 1 of 2 multiphase therapies (A: ~ 600 to 1500 Hz; B: ~ 300 to 600 Hz), completed a 2–3-day washout period, then received 11–12 days of multiphase therapy using existing leads and placement	Ongoing, prospective, multicenter, single-arm study with 24-month follow-up conducted in Australia with Ethics Committee approval Following a successful trial (≥ 50% reduction in overall VAS) with a Prospera SCS trial stimulator, patients are implanted with the permanent Prospera SCS system; post-implant follow-up includes in-person and remote management visits
Endpoints	Primary Endpoint 1: evaluate the change from baseline in NRS for overall pain for each multiphase SCS therapy Primary Endpoint 2: provide an inter-therapy comparison of change since baseline in NRS for overall pain	Primary Endpoint 1: responder rate (≥ 50% reduction in overall pain [VAS]) at the 6-month follow-up interval Primary Endpoint 2: primary safety information from permanent implant through the 6-month follow-up interval (AE-free rate)
Key results	77 participants were randomized to receive multiphase SCS and 65 completed the study Mean change from baseline to final in-office study visit in NRS overall pain score was −4.3 for therapy A and −4.7 for therapy B (both p < 0.0001) There were no statistically significant differences in NRS change between multiphase therapy A and B (p = 0.55)	Interim results for 22 participants at 12 months post-implant: Overall pain response rate at 12 months (≥ 50% pain reduction from baseline) = 82% Device- or procedure-related serious AEs = 1 (lead anchor failed, surgically resolved)

*AE* adverse event; *IRB* institutional review board; *NRS* numerical rating scale; *SCS* spinal cord stimulation; *VAS* visual analog scale

The BENEFIT-02 study evaluated the short-term safety and efficacy of two variants of RESONANCE stimulation (frequencies of approximately 600–1500 Hz and approximately 300–600 Hz) during extended SCS trials of patients with chronic back and leg pain [31]. Participants were required to have an overall pain numerical rating scale (NRS) score of at least 6 at baseline and were randomized to one of the two multiphase therapies. Following a successful commercial SCS trial, commercial external pulse generators (EPGs) were disconnected, and existing leads were connected to the investigational EPG; participants then entered a 2- to 3-day washout period. Multiphase stimulation (either the higher or lower frequency therapy) was activated after the washout period and delivered via the investigational EPG for 11 to 12 days. Among 65 participants with chronic low back and/or leg pain who completed the study, both multiphase therapies were associated with significant pain relief from baseline (both p < 0.0001) [31]. Notably, there was no statistically significant difference in pain relief between the higher frequency and lower frequency therapies (p = 0.55), suggesting that the lower frequency therapy could be used to promote battery usage and reduce potential overstimulation without compromising efficacy [31]. Furthermore, mean power usage of multiphase SCS therapy was lower than that of commercial SCS therapies for all participants receiving 300 to 600 Hz multiphase therapy (commercial SCS: 3.30 ± 0.82 milliwatts [mW], multiphase SCS: 0.60 ± 0.18 mW; p = 0.0016); particularly high power savings were observed for participants who only used 300 Hz multiphase therapy (commercial SCS: 3.44 ± 1.39 mW, multiphase SCS: 0.38 ± 0.08 mW; p = 0.024) [31]. Compared with commercial SCS therapies, the mean estimated vertebral coverage associated with both multiphase therapies was approximately doubled [31].

To build upon these clinical findings, BENEFIT-03 is an ongoing, prospective, multicenter trial conducted at up to 5 clinical sites in Australia to assess the long-term safety and efficacy of the Prospera SCS system and to evaluate the use of remote device management and the proactive care model [32]. Following consent and a successful trial, participants receive a permanent Prospera SCS System implant and undergo 24 months of follow-up. An interim analysis of 22 participants showed that overall pain response rate at 12 months (≥ 50% pain reduction from baseline) was 82% [33, 34]. Response rates for back pain (n = 21) and leg pain (n = 9) at 12 months were 76% and 89%, respectively, and high responder rate (≥ 80% pain reduction from baseline) at 12 months ranged from 48 to 78% [33, 34]. Most participants who used opioids at baseline were able to reduce their dose or eliminate opioid use entirely [33, 34]. Serious adverse events related to the device or procedure were rare, with a single event (failure of lead anchor, resolved by surgical replacement) reported at the time of interim analysis [33, 34].

Interim BENEFIT-03 data showed that proactive care enabled by remote monitoring allowed for SCS-related issues to be identified and corrected in a timely fashion [35, 36]. Results from an interim analysis indicated that automatic, daily, remote monitoring identified 115 events that qualified as a predefined proactive care trigger (eg, charging compliance, low device usage); following a trigger, mean time to address the SCS device-related issue was 2.2 days [35, 36]. In addition to the issues detected by automatic proactive care triggers, more complex issues can be identified by data review. In one case, the proactive care team identified an unintended program being used in a 79-year-old female on day 20 post-implant; during a real-time session, the study care team notified the participant, ensured the program was corrected, and provided education to the participant [37, 38]. This intervention occurred before the participant was negatively impacted by the unintended program. Subjective patient-reported outcomes (PROs) collected via at-home daily diary showed that in 19 implanted patients with 12-month interim data, mean days per week with mild/no pain (NRS ≤ 3) improved from 0.3 at baseline to 5.4 at 12 months [33, 34]. Trends in the daily patient-reported pain intensity scores were consistent with in-clinic assessments, and pain reduction corresponded with improvement from baseline in sleep quality as assessed via PROs [33, 34]. Surveys of participants with 12-month data available (n = 22) indicated that participants had positive experiences with remote device management and preferred it over traditional in-clinic follow-up [35, 36]. Clinician surveys indicated that remote device management with the Prospera SCS system benefited participants, improved chronic pain management, and reduced clinic staff burdens [35, 36]. Between implant and 12 months, clinicians estimated that remote device management saved participants an average of 3.9 in-person visits [35, 36]. To put this result into context, real-world evidence suggests that approximately 6 in-person visits are required each year to address SCS-related issues [29].

These early results from the BENEFIT-03 trial are promising and suggest that the Prospera SCS system, with its proprietary multiphase stimulation paradigm and remote device management capabilities, may provide effective pain relief while improving the efficiency of patient care and reducing burdens associated with in-person SCS visits. Further evaluation of full BENEFIT-03 study results is warranted to assess the role of the Prospera SCS system in the continuum of care for patients with chronic pain.

## Conclusions

The Prospera SCS system combines a proprietary multiphase stimulation paradigm with a proactive care model that allows for automatic, daily, objective, remote device monitoring and remote programming. Unlike other stimulation modalities, the multiphase paradigm known as RESONANCE delivers a continuous pattern of stimulation across a wider area of the spinal cord and at a lower electrical dose. In clinical studies, RESONANCE resulted in significant improvements in pain intensity from baseline. Early results from the ongoing BENEFIT-03 study suggest that proactive care, made possible through automatic daily remote device management, may identify and resolve SCS issues that may have otherwise gone undetected. Both patients and clinicians report positive experiences with remote device management, indicating that it is easy to use and reduces burdens. These advances represent new opportunities in the field of SCS to improve patient care.

## Data Availability

No datasets were generated or analysed during the current study.
